# An Account of a Case of Fever and Pain from Rheumatism Being Relieved by the Discharge of Electric Sparks from the Body

**Published:** 1807-10

**Authors:** Francis Johnson

**Affiliations:** Philadelphia


### *An Account of a Case of Fever and Pain from Rheumatism being relieved by the Discharge of electric Sparks from the Body.* By FRANCIS JOHNSON, Esq. near Philadelphia.

[ Extracted from the Philadelphia Medical Museum. ]

FOR many years, I had, from a variety of observations made upon my own *person* and *health*, conceived, that *good health* depended upon an exact equilibrium of the electric fluid being kept up within my system. I frequently found, on experiment, that if my pulse was languid, and my spirits depressed, my frame received with avidity (if I may so express myself) the electric fluid from any proper apparatus; but until about the years 1791 or 1792, I never contemplated that my health depended so much upon this fluid (if a redundancy of it was within me) being immediately ejected or drawn out.

In the fall of that period, for two successive days and nights, I felt feverish, with a total loss of appetite, a parched skin, with violent rheumatic pains in my head, breast, back, and stomach. Late in the evening of the last day, being frosty weather, I drew from my pocket a new silk handkerchief, which I had just purchased with some others of the like kind, then in another pocket; and as I entered my own door, being in great pain, I rubbed my head with it with much agitation and exertion, and immediately *perceived* and *heard* the electric sparks proceed from thence *most abundantly*. I then drew the handkerchief frequently through my hands from one extremity of it to the other, during all which time, *it*, or *I*, emitted a continued stream of *sparkling*, *crackling*, and *audible* fire.

As soon as I entered my common back parlour, or apartment which my family usually occupied during the fall and winter, I luckily met with a number of my friends and connections, some of whom, it is true, were *juvenile*, others however were of *maturer* years and of scientific knowledge, to whom I communicated this remarkable phenomenon, and whom I requested to make the like experiment with the *handkerchief* which I had done. Accordingly several of them did so; but none of them having fever, any chronic complaint, or listless or restless moments, but being all in perfect health, (I repeat it) *none of them* did, or could, produce the *like effect*.

An

An hour only elapsed, until I exhibited in the same neighbourhood, among three or four friends, the extraordinary manner (as already stated) in which I could emit the *electric fluid* through the medium of my *manual* operation on the silk handkerchief. At a small distance from the place of my last operation (if I may so call it), I met some others of my associates at the house of one of my most particular friends. At this plaee I felt *much better* in point of health, that is, my fever had considerably abated, the irritation of my nerves had subsided much, and my rheumatism (the principal cause of my complaint) was nearly removed; hence the *electric fluid* or *fire*, or by whatever other name it may be expressed, was not so vivid or luminous or abundant as it had been, yet still I performed the operation as heretofore stated, while those in *good health* around me could not do so. On my return home the same evening, I felt myself perfectly relieved from fever and pain, and with all my repeated endeavours could not emit one single spark of electricity. From the foregoing data, my corollary is—"*That good health depends much upon an exact equilibrium being kept up, of the electric fluid within our system.*" Whether any, or what *effects*, may be produced from the relation of the *facts* which are here stated, I know not. My sincere wish is, that they may prove useful to those persons whose study and business it is to collect and apply such facts for the improvement of the healing art, and the benefit of mankind.

